# Cushing's Disease Presenting with Functional Neurological (Conversion) Disorder

**DOI:** 10.1155/2023/1662271

**Published:** 2023-03-08

**Authors:** Sahar Ashrafzadeh, Maria Theresa Mariano, Saba Syed

**Affiliations:** ^1^David Geffen School of Medicine, University of California, Los Angeles, CA, USA; ^2^Department of Psychiatry and Biobehavioral Sciences, Olive View-UCLA Medical Center, Sylmar, CA, USA

## Abstract

While psychiatric manifestations are common in patients with Cushing's syndrome (CS), to our knowledge, there are no reported cases of CS presenting with functional neurological disorder (FND), a neuropsychiatric condition in which patients experience neurological symptoms, such as motor dysfunctions, sensory symptoms, speech disorders, or nonepileptic seizures, in the absence of neurological disease. Here, we report a case of a complex patient with Cushing's disease who presented with multiple FND symptoms including nonepileptic seizures, bilateral lower extremity paralysis, decreased finger flexion resulting in limited hand function, and stuttering. This case illustrates a rare psychiatric manifestation of CS presenting as multiple neurological complaints. Furthermore, we elucidate how a multidisciplinary treatment approach improved our patient's FND symptoms.

## 1. Introduction

Cushing's syndrome (CS) is a disorder resulting from exposure to excess cortisol. CS may be caused by exogenous corticosteroid use, adrenal tumors, bilateral adrenal hyperplasia, malignancies that produce ectopic adrenocorticotropic hormone (ACTH), or ACTH-secreting pituitary tumors. Cases of CS that are due to ACTH-secreting pituitary tumors are known as Cushing's disease. Patients with CS often present with insidious and nonspecific symptoms, including central adiposity, abdominal striae, thin skin, proximal myopathy, hirsutism, menstrual irregularity, fatigue, hypertension, and hyperglycemia [1]. Due to the rare occurrence of CS in the general population, hypervigilance is necessary since patients with CS are typically screened and diagnosed based on their clinical presentations rather than through routine screenings or lab tests [1].

CS is also frequently associated with various neuropsychological and cognitive changes. In some patients, psychiatric illness may be the presenting symptom [2–7]. Depression, irritability, and anxiety are among the most common psychiatric manifestations of CS [8]. In patients with CS who receive treatment, reductions in cortisol levels have been correlated with improvements in depression, anxiety, and somatic symptom scores [9]. However, recovery from psychiatric illness can be gradual and may take over 12 months after treatment of CS [10].

Functional neurological disorder (FND), also known as conversion disorder, is a neuropsychiatric condition in which a person experiences neurological symptoms, such as motor dysfunctions, sensory symptoms, speech disorders, or nonepileptic seizures, in the absence of neurological disease [11, 12]. While the etiology of FND is unclear, it is often associated with a psychological stressor, and recent research has identified that patients with FND demonstrate abnormal stress response and have elevated stress biomarkers levels, including cortisol [13].

We describe a case of a young man with Cushing's disease who presented to our hospital due to catatonia and multiple functional symptoms, including functional motor disorder and psychogenic nonepileptic seizures. Furthermore, we discuss how we used a multidisciplinary approach to treat his Cushing's disease, FND, and psychiatric symptoms during his hospitalization prior to pituitary resection surgery. To our knowledge, this is the first reported case of a patient with CS presenting with FND.

## 2. Case Report

A 28-year-old male with a chart history of paraplegia of unknown etiology for 6 months resulting in bedbound status, unspecified seizure disorder, untreated Cushing's disease, and hypertension presented to the hospital from a nursing facility due to increased frequency of seizure-like episodes.

On initial presentation, the patient was afebrile, hypertensive (160/101 mmHg), tachycardic (107 beats/minute), and tachypneic (22 breaths/minute), with 98% oxygen saturation on room air. The patient was not responding to verbal stimuli but would withdraw from pain. Physical exam was notable for dark striae on the abdomen, decreased strength in the upper extremities (rated 4/5 using the Medical Research Council scale for muscle strength [14]), and inability to lift the lower extremities against gravity. Laboratory studies were notable for hypokalemia (3.5 mEq/L) and thrombocytopenia (78,000/mL) but were otherwise unremarkable. Head CT was negative for acute intracranial findings. After receiving 2 mg lorazepam, he regained the ability to follow simple commands and to communicate, although with significant speech latency and hypoverbality. The medical team questioned the diagnosis of paraplegia based on movements elicited from both lower extremities. A brain MRI was obtained, but the patient experienced anxiety and seizure-like activity, and the results were limited by motion artifacts. A spine MRI was not obtained as the patient was able to move his extremities on physical exam. The patient underwent electroencephalogram (EEG) monitoring for three days and had multiple episodes of body, limb, and head shaking without epileptiform discharge. Additionally, the patient did not have incontinence, tongue biting, eye deviation, or postictal state with these episodes. Given these findings, neurologic workup was consistent with psychogenic nonepileptic seizures (PNES).

Psychiatry was consulted because the patient endorsed suicidal ideation. On initial psychiatric exam, the patient exhibited symptoms of catatonia with a Bush-Francis catatonia score of 12 [15]. Intravenous (IV) lorazepam 2 mg four times per day was given to treat catatonia, resulting in improvement in quantity of speech to full sentences. As his Bush-Francis catatonia score improved, the patient was transitioned from IV lorazepam to oral clonazepam 2 mg twice per day. After treatment of his catatonia, the patient was determined to be oriented to name and situation but not to date or location; formal cognitive assessments continued to be limited by significant speech latency and paucity of speech. The patient endorsed symptoms of depression and psychosis including depressed mood, anhedonia, poor appetite, psychomotor retardation, suicidal ideation, auditory hallucinations of talking or screaming voices, and visual hallucinations of people or “dark shadows” and was started on sertraline 200 mg daily and aripiprazole 25 mg daily. With treatment, the patient's oral intake and engagement with the treatment team improved. The seizure-like episodes continued to occur but decreased in frequency. However, the patient developed a new symptom of stuttering speech and continued to have residual symptoms of depression, including depressed mood, psychomotor retardation, and suicidal ideation as well as persistent auditory and visual hallucinations despite optimization of psychiatric medications. The treatment team was cautious to further increase the patient's antipsychotic medication dosage due to risk of maintaining or worsening his catatonia [16].

On further investigation, the psychiatry team learned that two years prior to admission, the patient's family noticed that the patient was developing physical changes including weight gain and striae. One year prior to admission, the patient appeared to have progressive extremity weakness and could not control the lower extremities. The patient was diagnosed with Cushing's disease in the outpatient setting but did not receive treatment due to concurrent onset and rapid worsening of psychiatric symptoms including depression with anhedonia, poor oral intake, memory impairment, and suicidal ideation resulting in a five-month admission to a med-psych hospital. During that hospitalization, the patient was no longer able to move his legs, resulting in bedbound status and a chart history of paraplegia. The patient also began having episodes of seizure-like activity and was started on valproic acid 1000 mg three times per day and levetiracetam 1500 mg twice per day but did not undergo formal epilepsy evaluation. The patient was not observed to have auditory or visual hallucinations during that hospitalization. The patient was eventually discharged to a nursing facility without any psychiatric medications and presented to our hospital three weeks later due to the seizure-like episodes increasing in frequency. Prior to these changes, the patient did not have any history of depression, substance use, or other psychiatric illness, and there was no known family history of psychiatric disorders. Given history obtained and physical appearance notable for central and facial adiposity, dorsal fat pad, striae, ecchymoses, and thinned skin, psychiatry advocated for workup of Cushing's disease.

**Figure 1:**
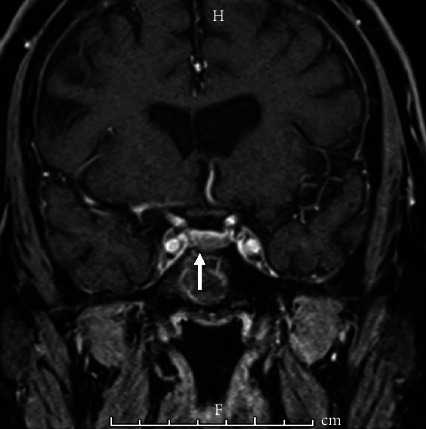
MRI of pituitary microadenoma consistent with Cushing's disease. Coronal T1-weighted enhanced MRI of the pituitary gland shows an anterior inferior pituitary lesion to the right of midline measuring approximately 6 × 6 × 3 mm.

An endocrinology consult guided further investigations. The patient's 24-hour urine cortisol level was extremely elevated to 1767.4 mcg/24 hr (normal: <50 mcg/24 hr), confirming a diagnosis of CS. In addition, serum cortisol values were elevated to 31.6 mcg/dL in the morning and 23.1 mcg/dL in the evening. Further workup revealed that plasma ACTH level was also increased to 119 pg/mL (normal: 6-50 pg/mL). An MRI of the sella turcica was ordered and showed a 6 × 6 × 3 mm pituitary microadenoma concerning for an ACTH-secreting tumor (Figure 1), resulting in a diagnosis of Cushing's disease. Treatment with cabergoline 2.5 mg three times per day on weekdays was initiated, and ketoconazole 250 mg three times per day was added given treatment resistance, leading to near-normal serum cortisol levels. Neurosurgery was consulted for resection of the pituitary microadenoma, which will be the definitive treatment.

Psychology, physical therapy, and occupational therapy were also involved. The patient regained hand function, including the ability to eat independently and to write using a pen. Furthermore, after being bedbound for eight months, the patient was once again able to stand up and take steps with assistance. Depressed mood, anhedonia, and suicidal ideation decreased as the patient's functional symptoms improved.

## 3. Discussion

CS is the result of chronic exposure to excess glucocorticoids, producing numerous physical and psychiatric changes. Approximately 70% of cases of CS are caused by a pituitary tumor that secretes ACTH, also known as Cushing's disease [17]. Additional causes of CS include adrenal adenomas and ectopic ACTH-producing tumors. A diagnosis of CS may be overlooked in patients presenting to the hospital with nonspecific psychiatric symptoms given that CS is often associated with more visible physical changes, including weight gain, fatty tissue deposition, muscle weakness, striae, thinned skin, easy bruising, acne, and hirsutism, as well clinical changes such as hypertension and glucose intolerance [1]. The neuropsychiatric manifestations of CS include depression, emotional lability, sleep disturbance, memory impairment, anxiety, panic disorder, mania, psychosis, and catatonia [8, 18, 19]. Psychiatric manifestations are very common among patients with CS, with 55-81% having depression, 12% having anxiety, 3-27% experiencing hypomania or mania, and 8% experiencing psychosis [18]. Furthermore, there have been reported cases of patients with CS initially presenting to the hospital due to psychiatric manifestations or catatonia [2–7]. Importantly, while there have been reported cases of depression, psychosis, and catatonia being cured after treatment of CS, studies have shown that neuropsychiatric symptoms may persist for at least one year in some patients despite medical or surgical treatment of CS leading to normalization of cortisol levels [10]. This may be attributed to anatomical and functional changes in the brain, including brain atrophy and loss of hippocampus volume, which can contribute to depression and cognitive impairment [8].

To date, there are no reported cases of CS presenting with FND, also known as conversion disorder. FND is a neuropsychiatric condition in which patients experience neurological symptoms, such as motor dysfunctions, sensory symptoms, speech disorders, or psychogenic nonepileptic seizures (PNES), in the absence of neurological disease [11]. In PNES, the individual experiences seizure-like behavior or a dissociative state in the absence of more subtle clinical signs of a seizure and without epileptiform discharge on EEG [20]. While its pathophysiology remains unclear, FND is often considered to be the physical manifestation of an unresolved stressor. In the conversion model of FND, it is thought that an individual represses emotions in reaction to traumatic or stressful events and converts emotional tension into physical symptoms as a protective defense mechanism [12]. However, contemporary neuroscience research suggests that the development of FND is multifactorial, involving both psychological and neurobiological mechanisms, including abnormal threat processing in the amygdala as well as differences in stress biomarker levels [12, 13]. Cortisol levels can serve as a biomarker of stress response, and some studies suggest that cortisol levels are dysregulated in patients with FND [21, 22]. One study found that patients with PNES had higher serum cortisol levels during their seizure-like episodes as well as in the afternoons and evenings when compared to healthy controls, although morning serum cortisol levels were similar between groups [22]. Another study found that patients with PNES had basal hypercortisolism when compared to healthy controls, regardless of seizure timing [23]. A meta-analysis and systematic review on endocrine biomarkers in patients with FND did not find a significant association between FND and cortisol levels but found that baseline ACTH may be dysregulated in patients with FND [24].

Interestingly, our patient experienced not just one manifestation of FND but rather an array of symptoms, including PNES, bilateral lower extremity paralysis resulting in prolonged bedbound status, decreased finger flexion resulting in limited hand function, and new-onset stuttering. Furthermore, the FND symptoms demonstrated a fluctuating course during the hospitalization. For example, the speech difficulties varied day-to-day, with the patient having significant stuttering and hypoverbality on some days but being able to speak in full sentences with decreased stuttering and improved rate of speech on other days. The patient's degree of finger flexion also varied day-to-day. FND symptoms can have a fluctuating course, with one study on 100 patients with functional movement disorder finding that 62% had fluctuations in the severity of their symptoms [25]. Additionally, it is not uncommon for patients with FND to have multiple functional symptoms or a history of previous functional symptoms [26]; one study found that 28 out of 100 patients with FND had mixed functional symptoms [27]. It is unknown if our patient's multiple functional symptoms arose due to untreated CS or from potential predisposing factors and external triggers. However, our patient's timeline suggests that his functional movement disorder and PNES arose concurrent with the development of physical and psychiatric manifestations of CS, and his functional stuttering arose during his hospitalization.

The constellation of our patient's various symptoms in conjunction with their fluctuations in severity made treatment challenging. Our patient initially presented with limited ability to engage with providers due to having catatonia that responded appropriately to first-line treatment of IV lorazepam. After the hypoverbality improved, the patient was able to communicate with the team about depression and hallucinations, enabling psychiatry and psychology to provide treatment with psychotropic medications and psychotherapy. Studies have shown that medications and psychotherapy are effective in helping CS patients with their psychiatric symptoms [18]. Furthermore, in addition to functional movement disorder, our patient had developed muscle weakness from CS and from eight months of bedbound status. Physical and occupational therapy teams helped the patient successfully regain strength and functioning of both upper and lower extremities.

Most importantly, since cortisol dysregulation can reduce activation of emotion-processing regions of the brain and lead to psychiatric and neurocognitive changes, Cushing's disease treatment was initiated [18]. The treatment for CS includes medical therapies such as adrenal enzyme inhibitors (e.g., ketoconazole), glucocorticoid receptor antagonists (e.g., mifepristone), the somatostatin analogue pasireotide, and the dopamine agonist cabergoline. However, for Cushing's disease, the first-line treatment is transsphenoidal surgery and/or pituitary irradiation. Among patients with a pituitary microadenoma similar to our case, transsphenoidal surgery is associated with a cure rate of up to 90% [28]. Given our patient's prolonged hospitalization in the setting of epilepsy workup, autonomic instability, catatonia, suicidal ideation, and barriers to discharge planning, we opted to initiate medical therapy of Cushing's disease while the patient was being medically and psychiatrically stabilized for surgery. The patient was initially started on cabergoline, but serum and 24-hour urine cortisol levels showed limited response. One study on patients with Cushing's disease found that six out of nine patients taking cabergoline had normalization of their 24-hour urine cortisol levels with the addition of ketoconazole [29]. After we initiated ketoconazole, our patient showed marked reduction in serum cortisol to near-normal levels.

Furthermore, our team provided patient education about FND and opted for an interdisciplinary approach involving physical therapy, occupational therapy, and psychotherapy to manage the patient's FND symptoms [30]. With this approach, there was improvement in PNES frequency, upper and lower extremity strength, hand function, and speech over the course of the hospital stay. A summary of treatments provided to our patient during the hospitalization is provided in Table 1. We anticipate our patient will have a positive outcome with potential improvement in FND and other psychiatric symptoms with transsphenoidal surgery.

This case report is limited by the unavailability of information on the outcome of the patient's pituitary resection surgery that was performed at an outside hospital as well as the lack of data on the improvement or progression of his FND after discharge. However, strengths of this case report include our ability to closely monitor his CS, FND, and psychiatric symptoms over the course of his two-month hospitalization as he received interdisciplinary care from multiple specialists and allied health services.

This case report illustrates a rare psychiatric manifestation of Cushing's disease presenting as FND or conversion disorder with mixed symptoms. Our patient experienced multiple FND symptoms that fluctuated in severity over time including PNES, bilateral lower extremity paralysis, limited finger flexion, and speech difficulties. Successful treatment of Cushing's syndrome with FND requires a multidisciplinary approach that may integrate internal medicine, psychiatry, neurology, endocrinology, neurosurgery, psychology, physical/occupational therapy, and other services depending on a patient's severity of illness and organ system involvement.

## Figures and Tables

**Table 1:** Multidisciplinary treatments provided to a patient with Cushing's disease presenting with functional neurological disorder.

Diagnosis	Treatment regimen
Catatonia	(i) Intravenous lorazepam (treatment) up to 2 mg four times per day (discontinued after improvement in catatonia) (ii) Oral clonazepam (maintenance) 2 mg twice per day
Depression	(i) Psychotherapy (ii) Sertraline 200 mg daily
Psychosis	(i) Psychotherapy (ii) Aripiprazole 25 mg daily
Cushing's disease	(i) Ketoconazole 250 mg three times per day (ii) Cabergoline 0.5 mg three times per day (weekdays only) (iii) Transsphenoidal pituitary microadenoma resection (planned procedure)
Psychogenic nonepileptic seizures	(i) Patient education (ii) Psychotherapy (iii) Antiepileptic medications were downtitrated (a) Levetiracetam was decreased from 1500 mg twice per day to 500 mg twice per day (b) Valproic acid 1000 mg three times per day was discontinued
Functional movement disorder	(i) Patient education (ii) Psychotherapy (iii) Physical therapy for leg strength, standing, and walking (iv) Occupational therapy for hand dexterity (e.g., writing, drawing, and stress ball)
Functional speech disorder	(i) Patient education (ii) Psychotherapy (iii) Speech therapy

## Abbreviations

ACTH: Adrenocorticotropic hormone
CS: Cushing's syndrome
EEG: Electroencephalogram
IV: Intravenous
FND: Functional neurological disorder
MDD: Major depressive disorder
mg: Milligram
PNES: Psychogenic nonepileptic seizures.
